# Research progress on the application of *Lacticaseibacillus rhamnosus* GG in pediatric respiratory diseases

**DOI:** 10.3389/fnut.2025.1553674

**Published:** 2025-02-21

**Authors:** Yang Liu, Yan Chen, Huijuan Liao, Shijie Sun, Xiaohu Zhang, Liang Xie, Hanmin Liu

**Affiliations:** ^1^Department of Pediatric Pulmonology and Immunology, West China Second University Hospital, Sichuan University, Chengdu, China; ^2^Key Laboratory of Birth Defects and Related Diseases of Women and Children, Sichuan University, Ministry of Education, Chengdu, China; ^3^NHC Key Laboratory of Chronobiology (Sichuan University), Chengdu, China; ^4^The Joint Laboratory for Lung Development and Related Diseases of West China Second University Hospital, Sichuan University and School of Life Sciences of Fudan University, West China Institute of Women and Children's Health, West China Second University Hospital, Sichuan University, Chengdu, China; ^5^Sichuan University-The Chinese University of Hong Kong Joint Laboratory for Reproductive Medicine, West China Second University Hospital, Sichuan University, Chengdu, China; ^6^Development and Related Diseases of Women and Children Key Laboratory of Sichuan Province, West China Second University Hospital, Sichuan University, Chengdu, Sichuan, China

**Keywords:** *Lacticaseibacillus rhamnosus* GG, pediatrics, respiratory diseases, gut-lung axis, probiotics, immunomodulation, microbiota

## Abstract

Respiratory diseases are a leading cause of morbidity in children globally, with significant healthcare costs. The overuse of conventional treatments like antibiotics has raised concerns about antibiotic resistance and side effects. *Lacticaseibacillus rhamnosus* GG (LGG), one of the most extensively studied probiotics, has gained attention as a potential adjunct therapies due to their ability to modulate the gut microbiota and immune responses. This review aims to assess the effectiveness of LGG in managing pediatric respiratory diseases, including respiratory tract infections (RTI), cystic fibrosis (CF), and asthma. Clinical trials suggest LGG can reduce the incidence and severity of RTI, improving CF symptoms, and enhancing quality of life in children. However, evidence for its benefits in asthma remains inconclusive. Its mechanisms include modulating immune responses, enhancing gut barrier function, and maintaining a microbial homeostasis via the gut-lung axis. Existing studies are often limited by small sample sizes, heterogeneity in intervention protocols, and short follow-up periods. Emerging technologies and novel formulations, hold promise for unraveling the complex interactions among LGG, the gut-lung axis, and respiratory health. These advancements could pave the way for personalized probiotic therapies, highlighting the potential of LGG as a cost-effective, adjunctive therapy for pediatric respiratory diseases. This review underscores the broader significance of integrating LGG into pediatric healthcare, while calling for future research to overcome current limitations, optimize clinical protocols, and explore innovative therapeutic strategies.

## Graphical abstract

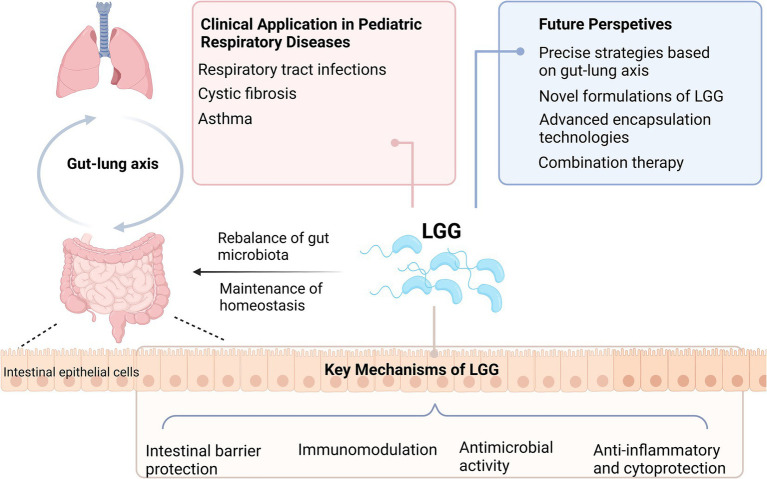

## 1 Introduction

Respiratory diseases are among the most common health issues affecting children worldwide, contributing significantly to morbidity and healthcare costs (1, 2). While conventional treatments such as antibiotics and bronchodilators are widely used, their overuse has raised concerns about antibiotic resistance and adverse side effects. Consequently, there is growing interest in alternative or adjunctive therapies, such as probiotics, which have shown promise in modulating host immunity and maintaining microbial homeostasis.

Probiotics, are living microorganisms that, when provided in sufficient quantities, offer beneficial effects on the health of the host (3). They primarily achieve their benefits through interactions with the gut microbes, enhancing the gut barrier function, and modulating systemic immune responses. Among the emerging concepts in microbiome research, the gut-lung axis has gained significant attention in recent years (4–6). This bidirectional communication pathway between the gut and the lungs highlights how gut-derived factors can influence pulmonary immunity and inflammation through microbial metabolites, cytokine signaling, and immune cell trafficking. An imbalance in the gut microbial communities, known as dysbiosis, has been linked to increased susceptibility to respiratory infections and chronic respiratory diseases, emphasizing the potential role of probiotics in restoring microbial equilibrium and improving respiratory health (7).

*Lacticaseibacillus rhamnosus* GG (LGG), previously known as *Lactobacillus rhamnosus* GG, is one of the most extensively studied probiotic strains. Reclassified in 2020 due to advancements in genomic analysis (8). LGG has demonstrated remarkable resilience in the gastrointestinal tract and the ability to adhere to intestinal epithelial cells, thereby exerting beneficial effects on the host’s immune system. Recent studies have suggested that LGG may play a role in reducing the incidence and severity of respiratory infections by enhancing mucosal immunity and mitigating inflammation. This article reviews the application of LGG in managing pediatric respiratory diseases, focusing on its mechanisms of action, clinical efficacy, and potential as a preventive and therapeutic agent. By synthesizing evidence from clinical trials and recent meta-analyses, we aim to provide a comprehensive overview of LGG’s role in pediatric respiratory health and identify areas for future research (see Graphical abstract).

## 2 Overview of LGG

### 2.1 Introduction to LGG

*Lacticaseibacillus rhamnosus* GG was first isolated and named by American professors Sherwood Gorbach and Barry Goldin from healthy humans in 1983. It is a naturally occurring strain of *Lacticaseibacillus*, a beneficial bacterium commonly found in the human gut. In 2020, *Lactobacillus rhamnosus* GG (LGG) was reclassified as *Lacticaseibacillus rhamnosus* GG following a comprehensive genomic analysis of the *Lactobacillus* genus. This reclassification highlights the genetic diversity within the genus and provides a more accurate taxonomic framework for studying probiotics. LGG is known for its ability to survive the harsh conditions of the stomach and intestines due to its high tolerance to acid and bile. This allow it to reach the intestines alive and exert its beneficial effects, unlike many other probiotics that are already killed by the effects of stomach acid and bile before entering the intestines.

### 2.2 Key features and mechanisms of LGG

Over the years, many studies have explored the mechanism of action of LGG (9), mainly including the following points:

#### 2.2.1 Adhesion and intestinal barrier protection

LGG exhibits strong adhesion capabilities, which are essential for its colonization and beneficial effects in the gut. This is facilitated by specific surface proteins, such as LGG-0186 and SpaCBA pili (10–12). These proteins enable LGG to bind effectively to intestinal epithelial cells, thereby outcompeting pathogenic bacteria for attachment sites. Additionally, LGG produces exopolysaccharides (EPS) rich in galactose, which form a protective shield against complement-mediated lysis (13, 14). This EPS layer not only protects LGG cells but also contributes to the overall stability of the gut microbiota. Moreover, LGG has been shown to normalizes intestinal permeability and improves intestinal barrier function (15).

#### 2.2.2 Immunomodulation

LGG exerts significant immunomodulatory effects, which are central to its health benefits. It stimulates nonspecific immune responses, including IgA, IgG, and IgM, which play crucial roles in defensing against pathogens (16, 17). LGG modulates cellular responses through its soluble factors, with effects that are cell-type and context-dependent. For instance, in dendritic cells (DCs), LGG can either enhance or suppress the expression of interleukin-6 (IL-6), depending on the microenvironment and overall health status. In inflammatory settings, it may promote IL-6 production to trigger defense mechanisms. By contrast, under anti-inflammatory or homeostatic conditions, it can indirectly suppress IL-6 secretion by inducing anti-inflammatory cytokines like interleukin-10 (IL-10) and Transforming Growth Factor-*β* (TGF-β). In macrophages, LGG typically reduces the production of pro-inflammatory cytokines such as Tumor Necrosis Factor-*α* (TNF-α) and interleukin-12 (IL-12) while boosting the secretion of anti-inflammatory cytokines like IL-10 and TGF-β (18–20). This dual action helps to fine-tune the immune response, preventing excessive inflammation while ensuring effective pathogen clearance.

Furthermore, LGG influences the balance between T helper cell 17 (Th17) and regulatory T cells (Treg cell) by regulating dendritic cell function (20–22). Through these mechanisms, LGG can reduce the overreaction of CD4 + T cells, a phenomenon often associated with autoimmune and inflammatory diseases (21, 23). Additionally, LGG promotes the secretion of IL-23 by dendritic cells in certain inflammatory contexts, which further facilitates the activation of type 3 innate lymphoid cells (ILC3) and enhance the production of IL-22. IL-22 plays a critical role in maintaining intestinal epithelial barrier integrity and promoting antimicrobial defense (24, 25).

#### 2.2.3 Antimicrobial activity

LGG produces lactic acid and various antimicrobial peptides, which inhibit the adhesion and growth of pathogens such as *Salmonella* and *E. coli* (12, 13). These antimicrobial substances create an unfavorable environment for pathogens, reducing their ability to colonize the gut. LGG’s competitive exclusion mechanism further prevents pathogen overgrowth by outcompeting them for nutrients and attachment sites on the intestinal epithelium.

#### 2.2.4 Anti-inflammatory and cytoprotection

LGG secretes soluble proteins such as main secreting protein (Msp), Msp/p40 and Msp1/p75, which reduce apoptosis in intestinal epithelial cells and protect the gut epithelial barrier (26, 27). These proteins help maintain the integrity of the intestinal lining, preventing the loss of barrier function that can lead to inflammation and infection. Additionally, LGG regulates the activity of pro-inflammatory cytokines like TNF-*α* and IL-10, thereby modulating the inflammatory responses and promoting a more balanced immune environment (28, 29).

## 3 Application of LGG in children with respiratory diseases

Given the intricate gut-lung axis, where the gut microbiota can influence respiratory health, LGG’s immunomodulatory and anti-inflammatory properties make it a promising candidate for managing pediatric respiratory diseases. With this foundation, we now delve into the specific applications of LGG in addressing respiratory issues in children. Table 1 summarized the random clinical trials (RCT) of LGG in pediatric respiratory disease.

**Table 1:** A summary of randomized clinical trials on LGG in pediatric respiratory diseases.

Research area	First author	Country/Region	Study population	Methods	Dosage of LGG	Duration of intervention/follow-up	Outcomes	Findings	Limitations	Reference
RTI	Luoto, 2014	Finland	94 preterm infants (gestational age 32 + 0 to 36 + 6 weeks; birth weight > 1,500 g)	RCT: administered prebiotics, LGG, or placebo, capsules mixed with a small amount of breast milk or formula	1 × 10^9^ CFU/day for 1 to 30 days and 2 × 10^9^ CFU/day for 31 to 60 days	3–60 days post-birth/follow-up for 12 months	The incidence of clinically defined virus-associated RTI episodes confirmed from nasal swabs; the severity and duration of RTI	RTI incidence significantly reduced; and rhinovirus infections significantly decreased in prebiotics and LGG group	Limited sample size; specificity of the study population (preterm infant); long-term effects not assessed	(30)
RTI	Kumpu, 2012	Finland	523 children in daycare centers (LGG group: *n* = 251, age = 4.0 ± 1.3 years; placebo group: *n* = 250, age = 4.0 ± 1.4 years)	RCT: consumed milk containing LGG or regular milk	10^8^ CFU/day	28 weeks From Oct to Apr	Rate of respiratory illness; gastrointestinal symptoms	Days with respiratory symptoms significantly reduced in the LGG group	Potential intake of lactobacillus in the control group; reliance on symptom reporting; unclear dose–response relationship	(31)
RTI	Hojask, 2010	Croatia	281 children in daycare centers (LGG group: *n* = 139, mean age = 51.9 months; placebo group: *n* = 142, mean age = 53.6 months)	RCT: consumed fermented milk containing LGG or placebo	1 × 10^9^ CFU in 100 mL milk /day	3 months (From Nov to Feb)	Risk of upper respiratory infection; risk of gastrointestinal infections	Risk of upper RTI reduced; days with respiratory symptoms significantly decreased	Short study duration; unclear causes of infection; low number of severe infection cases; incomplete control of another probiotic intake	(33)
RTI	Hojask, 2010	Croatia	742 hospitalized children (LGG group: *n* = 376, mean age of 9.9 ± 5.1 years; placebo group: *n* = 366, mean age = 10.6 ± 5.0 years)	RCT: consumed fermented milk containing LGG or placebo	1 × 10^9^ CFU in 100 mL milk /day	From admission to discharge, with an average hospital stay of 4–5 days. /Follow-up for 7 days after discharge	Risk of upper RTI; risk of gastrointestinal infections	Risk of gastrointestinal infections and upper RTI reduced	Inability to include infants, unknown causes of infection, high NNT value, single-center design, strict exclusion criteria, and lack of long-term follow-up	(34)
RTI	Hatakka, 2001	Finland	571 healthy children in daycare centers (LGG group: *n* = 282, age = 4.6 ± 1.5 years; placebo group: *n* = 289, age = 4.4 ± 1.5 years)	RCT: consumed milk containing LGG or placebo milk	1.3–2.6 × 10^8^ CFU/day based on 5–10 × 10^5^ CFU/mL milk ×260 mL/day	7 months over winter	Sick leave days, risk of respiratory and gastrointestinal symptoms or days with any illness; infections with complications; and antibiotic treatments	Sick leave days, infections with complications and antibiotic use decreased	Uneven age distribution; potential intake of lactobacillus in the control group; unknown long-term effects	(32)
CF	Bruzzese,2014	Italy	22 CF children aged 2–9 years (LGG group: *n* = 10, placebo group: *n* = 10)	RCT: oral administration of LGG	6 × 10^9^ CFU/day	4 weeks	Gut microbiota composition; intestinal inflammation measured by CLP levels	Partial restoration of gut microbiota composition; reduced CLP levels	Small sample size; single-center study; short-term intervention and follow-up	(37)
CF	Jafari, 2013	Iran	37 CF children (LGG group: *n* = 20, mean age = 5.36 years; placebo group: *n* = 17, mean age = 5.5 years)	RCT: administered multi-strain probiotic capsules containing LGG	2 × 10^9^ CFU/d	1 months / follow-up to 6 months	Quality of life score measured by PedsQL™4.0 questionnaire; rate of pulmonary exacerbation	Pulmonary exacerbation rate reduced within 3 months; QOL score significantly improved at 3 months	Difficult to isolate LGG contribution; effects may be temporary	(38)
CF	Bruzzese,2007	Italy	38 CF patients (Group A: *n* = 19, mean age = 12.8 years; Group B: *n* = 19, mean age = 13.7 years)	Prospective randomized controlled crossover study. Group A: LGG 6 month shift to ORS 6 months; Group B: ORS 6 month shift to LGG 6 months	6 × 10^9^ CFU/day	12 months	Incidence of pulmonary exacerbations and of hospital admissions, FEV1, and modifications of body weight; serum IgA, IgG, IgM	LGG significantly reduced pulmonary exacerbations and hospitalization rates	Small sample size; single-center study; potential bias in crossover design; baseline differences in lung function	(36)
Asthma related	Cabana, 2017	United States	184 high-risk infants (at least one parent with asthma, LGG group, *n* = 92; placebo group, *n* = 92)	RCT with LGG (10 billion CFU/day) + inulin vs. inulin only; randomized, double-blind, parallel-arm design	1 × 10^10^ CFU/day	6 months intervention/follow-up to 5 years	Cumulative incidence of eczema at 2 years; incidence of asthma and allergic rhinitis at 5 years	No significant reduction in eczema or asthma incidence	Small sample size; high breastfeeding rates may overshadow LGG effects	(52)
Asthma related	Jerzynska, 2016	Poland	100 children, ages 5–12 years, with grass pollen allergy and allergic rhinitis (12 with concomitant asthma; SLIT-placebo group, *n* = 24; SLIT-vitamin D group, *n* = 21; SLIT-LGG group, *n* = 20; control group, *n* = 17)	RCT: 5-grass SLIT tablets combined with LGG, vitamin D, or placebo	3 × 10^9^ CFU/day	5 months intervention during pollen season	Symptom-medication score, lung function (FEV1, FEV1%VC), FeNO, CD4^+^CD25^+^Foxp3^+^ induction, TLR-positive cells, TGF-β1 production	LGG group showed better clinical and immunologic response compared to SLIT alone or with vitamin D	Small sample size; no SLIT placebo for control group; limited asthma cases; no long-term follow-up	(51)
Asthma related	Ou,2012	Taiwan	191 pregnant women and their high-risk infants (LGG group, *n* = 95; placebo group, *n* = 96)	RCT: Pregnant women took LGG or placebo starting from the 24th week of pregnancy; postpartum, LGG was continued for mothers or directly administered to infants	1 × 10^10^ CFU/day	From 24th week of pregnancy to delivery; 6 months postpartum/follow-up to 36 months	The cumulative prevalence of sensitization and developing of allergic diseases; maternal allergic symptom score and plasma immune parameter	Improvement in maternal allergic symptoms but no significant effect on asthma in children.	Small sample size; limited long-term follow-up; study restricted to high-risk populations	(49)
Asthma related	Boyle, 2011	Australia	250 pregnant women with high-risk infants (LGG group, *n* = 125; placebo group, *n* = 125)	RCT: Pregnant women took LGG or placebo starting from the 36th week of pregnancy until delivery	1.8 × 10^10^ CFU/day	From the 36th week of pregnancy to delivery/follow up to 1 year	Incidence of eczema, allergic sensitization, IgE-associated eczema, gastrointestinal and respiratory symptoms.	No significant reduce in eczema and asthma prevalence or affect immune markers.	LGG use was limited to the prenatal period; insufficient sample size to detect small effects	(50)
Asthma related	Rose, 2010	Germany	131 children (6–24 months old) with recurrent wheeze and a first-degree family history of atopic disease (LGG group, *n* = 65; placebo group, *n* = 66)	RCT: LGG (1 × 10^10^ CFU) or placebo for 6 months; double-blind, prospective study	1 × 10^10^ CFU/day	6 months intervention/6 months follow-up	severity scoring of atopic dermatitis (SCORAD) and asthma-related clinical events	No significant differences in asthma-related events or atopic dermatitis; reduced sensitization to aeroallergens, effects persisted 6 months after intervention	Small sample size; reliance on caregiver-reported symptoms; no long-term follow-up available	(53)
Asthma related	Kuitunen, 2009	Finland	1,223 pregnant women and their high-risk infants (at least one parent with allergies) (LGG group, *n* = 445; placebo group, *n* = 446)	RCT: Pregnant women received combined probiotics including LGG or placebo from 36 weeks of gestation; infants received the same formulation from birth to 6 months	5 × 10^9^ CFU LGG/day (combined with other strains)	From 36 weeks of pregnancy to 6 months postpartum; follow-up to 5 years	Cumulative incidence of allergic diseases (eczema, food allergy, asthma, allergic rhinitis) and IgE sensitization	No significant reduction in overall allergic diseases or IgE sensitization at 5 years; significant reduction in IgE-associated allergic diseases in cesarean-delivered children	Subgroup analysis showed benefit only for cesarean-delivered children; potential confounding factors such as mode of delivery and breastfeeding duration	(47)

RCTs, Randomized controlled trials; LGG, Lacticaseibacillus rhamnosus GG; RTI, Respiratory tract infections; CF, Cystic fibrosis; CFU, colony forming units; FEV1, Forced expiratory volume in 1 s; FEV1%VC, Forced expiratory volume in 1 s as a percentage of vital capacity; ORS, oral rehydration solution; CLP, calprotectin; NNT, number needed to treat; QOL score, quality-of-life score; SLIT, sublingual immunotherapy; FeNO, fractional exhaled nitric oxide concentration.

### 3.1 Respiratory tract infections

Increasing clinical evidence suggests that LGG have a positive effect on RTI. A Finnish RCT study (30) included 94 preterm infants administrated oral prebiotics, LGG, or placebo between 3 to 60 days after birth. The results showed that the incidence of RTI was significantly lower in infants receiving prebiotics or LGG. Additionally, the incidence of rhinovirus-induced episodes (accounting for 80% of all RTI episodes) was notably reduced in these groups. However, the study focused on preterm infants born at 32 to 36 weeks of gestation. This specific population has a different immune system and gut microbiota composition compared to full-term infants, making the findings difficult to generalize to full-term infants or other age groups.

Another Finish study included 523 children aged 2–6 years and lasted for 28 weeks. (31) LGG intake was confirmed via fecal samples. Children who provided fecal samples at both the beginning and end of the study were classified as completed cases. In the completed cases, the number of respiratory symptom days in the LGG group was significantly reduced (4.71 days vs. 5.67 days). Hatakka et al. (32) studied 571 children aged 1 to 6 years who were given milk containing LGG or placebo milk for 7 months. The LGG group had fewer sick leave days (4.9 days vs. 5.8 days), lower rate of respiratory infections, fewer complications and less antibiotic use. Hojsak et al. (33, 34) also reported similar findings. In two studies conducted on healthy children attending daycare centers and hospitalized children, respectively, they observed a significant reduction in the risk of upper respiratory tract infections in children receiving LGG.

A meta-analysis (35) included 15 RCTs involving 5,121 children (aged 3 months to 7 years) in daycare centers. Among them, a pooled analysis of the above 3 RCTs, involving a total of 1,295 participants, showed that LGG significantly shortened the duration of RTIs (95% confidence interval [CI] −1.46; −0.09). However, another analysis involving 343 participants showed that *Bifidobacterium animalis subsp. lactis* BB-12 had no effect on the duration of RTIs or absenteeism.

Despite the promising results, these studies have several limitations such as small sample sizes, heterogeneous interventions (e.g., LGG capsules vs. fermented dairy products), and short follow-up periods, which limit the evaluation of LGG’s long-term protective effects. Additionally, study populations often vary in living environments, infection risks, and baseline health conditions.

### 3.2 Cystic fibrosis

Research on LGG in CF began as early as 2007. An Italian randomized, placebo-controlled crossover study found that 19 patients receiving LGG treatment had significantly reduced pulmonary exacerbations and hospitalization rates compared to those treated with oral rehydration solution (36). The research team later conducted a study on 22 children with CF. They found that oral LGG treatment significantly increased the intestinal *Bacteroides* counts in CF children, partially restored the composition of the gut microbiota, and reduced fecal calprotectin (CLP), a marker of intestinal inflammatory status (37). Another RCT (38) in Iran recruited 37 CF children who were treated with a multi-strain probiotic capsule containing LGG for 1 month. Using the PedsQL 4.0 questionnaire, the parents of the children reported a significantly lower rate of pulmonary exacerbations within 3 months after the intervention (*p* < 0.01). Additionally, the average total quality-of-life score showed significant improvement at the third month (*p* = 0.01) but not significant at 6 month, suggesting temporary benefits. Continuous intake may result in more stable improvements in quality of life. Since it was a multi-strain probiotic, it is difficult to individually evaluate the contribution of LGG to CF patients in this study.

Coffey et al. (39) conducted a systematic review of 17 trials (12 RCTs, 464 participants) on LGG and other probiotics for CF. Among these, 8 trials included only children, while four trials included both children and adults. Four trials specifically used LGG. The analysis showed that LGG may reduce intestinal inflammation but have limited effects on pulmonary exacerbations, lung function, or growth.

While research on LGG in CF started early, but to date, studies in children remain limited, with most involving small sample sizes. For example, one study included only 19 children in LGG group, resulting in insufficient statistical power that may fail to detect small but clinically significant effects. Additionally, some studies used multi-strain probiotic formulations, making it difficult to determine the specific effects of LGG. Existing studies have also primarily focused on short-term intervention effects, lacking long-term effects of LGG on key indicators such as lung function and nutritional status.

### 3.3 Asthma and related allergic disease

In children with asthma, gut microbiota dysbiosis is commonly observed (40–42). Therefore, multiple studies have explored the preventive and therapeutic effects of various probiotics, including LGG, on asthma and related allergic diseases (43–47).

Prenatal and perinatal supplementation with LGG and other probiotics appears to have minimal preventive effects (48).Ou et al. included 191 pregnant women who received LGG starting from the mid-pregnancy. While LGG reduced the severity of allergic diseases in mothers, it did not lower the incidence of allergic sensitization or allergic diseases in children (49). Boyle et al. had similar findings, but they only provided LGG to pregnant women during pregnancy without postnatal supplementation (50).

The effects of direct administration of LGG to children with asthma are also inconsistent. Jerzynska et al. reported significant improvements in lung function, regulatory T cells induction, and fractional exhaled nitric oxide concentration (FeNO) in children with allergic rhinitis sensitive to grass pollen who received LGG supplementation (51). However, Cabana et al. found different results. In high-risk infants with at least one parent with asthma and eczema, LGG supplementation within the first 6 months after birth did not prevent eczema or asthma by age 2. By age 5, the cumulative incidence of asthma in the LGG group showed a decreasing trend compared to the control group (9.7% vs. 17.4%), but the difference was not statistically significant (52). Similarly, Rose et al. found no significant differences in atopic dermatitis or asthma-related events. The study focused on children with at least two wheezing episodes and a family history of first-degree atopic disease who received LGG (53). Furthermore, in a subgroup of children previously sensitized to allergens, asthma symptoms showed a slight worsening.

The inconsistent results of LGG may partly stem from the multifaced nature of asthma pathogenesis, involving intricate interactions among genetic, environmental, and immunological factors. Variability in study design may also contribute to these discrepancies.

It is worth noting that in the treatment of asthma, researchers tend to favor the use of multi-strain probiotics in recent years. These combinations may also enhance gut microbial diversity and stability, which are critical for immune homeostasis. Stronger evidence supports the therapeutic effects of multi-strain probiotics, including combinations of *Lactobacilli* and *Bifidobacteria*, on asthma (44, 45, 54). As asthma involves complex interactions between the immune system and the gut-lung axis, it is necessary to further explore not only how different probiotic strains exert their specific mechanisms through this pathway but also how these strains interact synergistically to enhance and amplify their collective effects, particularly in the context of advancing microbiome research.

### 3.4 Challenges and findings in determining optimal dosages of LGG

The dosage of LGG is one of the key factors influencing their clinical effects, yet significant gaps remain on its use in pediatric respiratory diseases. Current studies report a wide range of dosage, typically between 10^8^–10^10^ colony-forming units (CFU) /day, reflecting the lack of consensus on optimal amounts. For example, LGG was generally administered at 10^8^–10^9^ CFU/day for RTI, 2 × 10^9^–6 × 10^9^ CFU/day for CF, and 3 × 10^9^–10^10^ CFU/day for asthma prevention or treatment.

However, there is no clear dose–response relationship established, making it difficult to determine the optimal dosage for different populations. Several gaps remain in understanding the optimal dosages of LGG. Firstly, the majority of studies have focused on specific age groups or health conditions, such as preterm infants or children in day care centers. This limits the generalizability of findings to other pediatric populations like adolescents or those with chronic respiratory conditions. Secondly, the duration of LGG intervention has varied across studies, from weeks to months. This variation makes it difficult to determine the long-term effects of LGG. Thirdly, the form of LGG administration (e.g., in milk, fermented products, or capsules) may influence its bioavailability and efficacy.

Future research should prioritize multicenter, large-scale, and long-term RCTs, explore LGG’s synergy with other probiotics, and develop personalized dosage recommendations based on microbiome profiles. Addressing these gaps will help optimize LGG’s use in managing RTI, CF, asthma, and other pediatric conditions.

### 3.5 Possible mechanisms of LGG specifically in respiratory diseases

Although research on LGG’s effects in respiratory diseases is still limited, recent studies have begun to shed light on its potential mechanisms, primarily mediated via the gut-lung axis (4–6).

Through its well-documented immunomodulatory properties, as described earlier, LGG exerts systemic effects that extend to the respiratory system. For example, studies have shown that Th17 cells in the gut can migrate to the lungs through the lymphatic system and bloodstream, where they contribute to immune regulation and pathogen defense (6). By promoting the balance of Th17 and regulatory T cells (Treg), LGG may indirectly influence pulmonary immune responses and reduce excessive inflammation.

LGG has also been shown to modulate the gut microbiota by increasing the abundance of beneficial bacteria, such as *Bifidobacteria* and *Lactobacilli* while reducing dysbiosis (10, 55). This rebalancing enhances the production of short chain fatty acids (SCFAs). SCFAs have been shown to reduce airway inflammation and improve lung function by modulating the activity of immune cells, including macrophages and dendritic cells (56–60).

Additionally, LGG enhances the integrity of the intestinal barrier by upregulating tight junction proteins such as occludin and claudin (15, 29). This prevents the translocation of endotoxins and pro-inflammatory molecules into the bloodstream, thereby reducing systemic inflammation that could exacerbate respiratory conditions.

**Figure 1:**
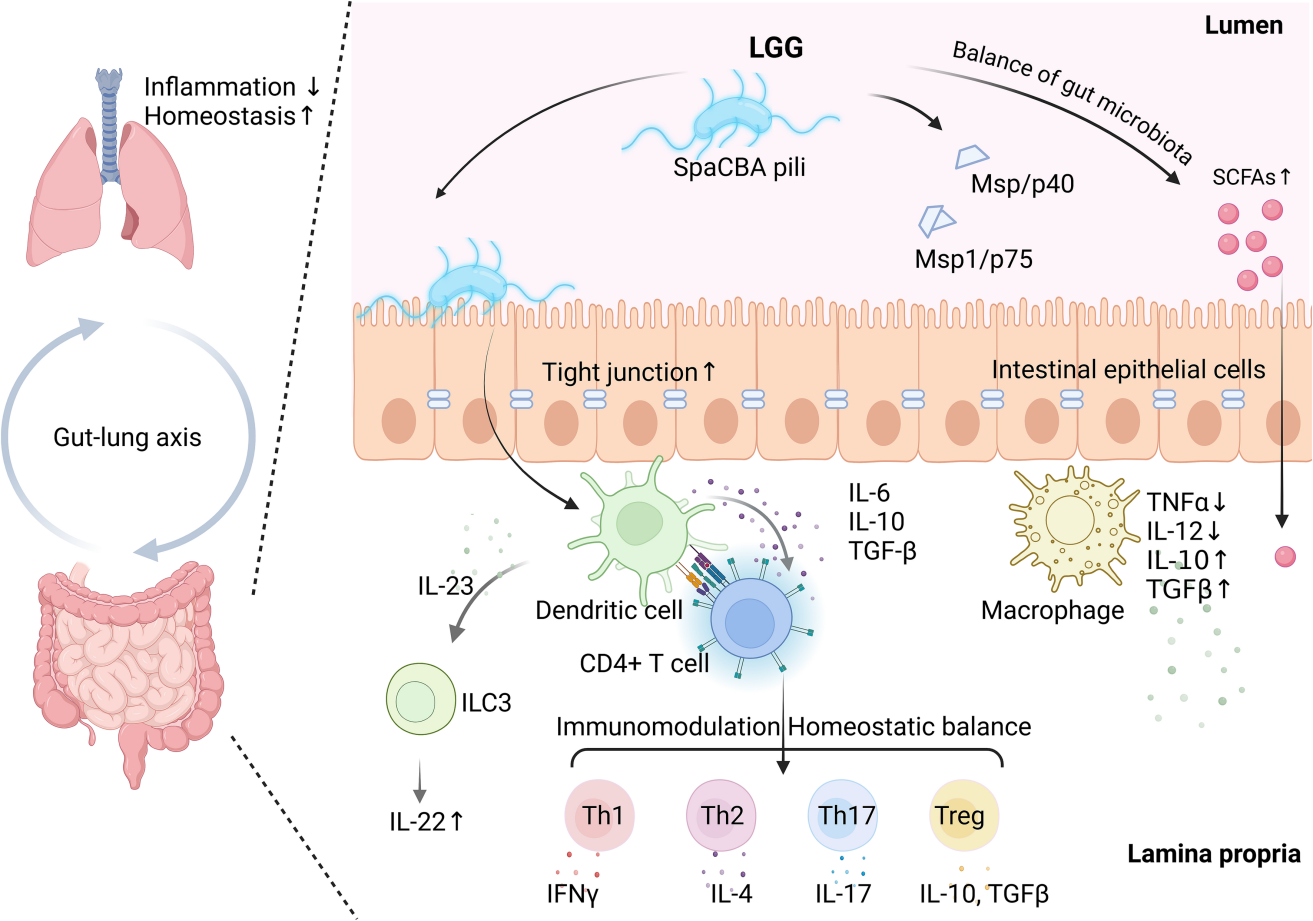
Schematic illustration of key mechanisms of *Lactobacillus rhamnosus* GG (LGG) in gut-gung axis and respiratory health. LGG can interacts with intestinal epithelial cells via SpaCBA pili. It can also enhance tight junction integrity and promote gut barrier function. Additionally, LGG helps balance gut microbiota and indirectly supports short-chain fatty acids (SCFAs) production by improving the gut environment and promoting the growth of SCFA-producing bacteria. Its metabolites, such as main secreting protein (Msp), Msp/p40 and Msp1/p75, play a role in maintaining epithelial health and modulating immune responses. These interactions in turn influence immune responses in the lamina propria. Macrophages are activated to secrete anti-inflammatory cytokines (IL-10, TGF-*β*) and reduce pro-inflammatory cytokines (TNF-*α*, IL-12). Dendritic cells further stimulate CD4+ T cells and innate lymphoid cells (ILC3), promoting IL-22 production and maintaining homeostatic balance among Th1, Th2, Th17, and Treg cells. This balance supports systemic immune modulation and highlights the gut-lung axis as a key pathway through which LGG promotes broader respiratory health benefits. Created in BioRender.com.

Figure 1 illustrates the key mechanisms of LGG in gut-lung axis and respiratory health.

### 3.6 Safety and adverse reactions

Since 1990, the LGG has been used worldwide as an ingredient in food and dietary supplements, with no adverse events reported to date. LGG is generally considered safe for most populations, including infants, children, and adults. It is well-tolerated and has a low risk of side effects, although some individuals may experience mild gastrointestinal symptoms such as bloating or gas.

## 4 Conclusion and future perspectives

LGG is one of the well-studied probiotics with significant potential as a safe and effective adjunct therapy for pediatric respiratory diseases. Clinical evidence suggests that LGG reduces the incidence and severity of RTI, shortens symptom duration, and lowers antibiotic use in children. It may also alleviate certain symptoms associated with CF and asthma, though evidence remains limited and varies by population. These benefits are attributed to its ability to modulate immune responses, enhance gut barrier function, and maintain a healthy microbiota balance, likely mediated through the gut-lung axis.

However, there have been relatively few groundbreaking findings regarding LGG in the field of pediatric respiratory diseases over the past 5 years. This trend may be attributed to several factors. Firstly, research in pediatric populations faces unique challenges, including ethical considerations, variability in immune development, and difficulties in long-term follow-up. Secondly, the research focus in probiotics field has shifted toward exploring multi-strain formulations and personalized microbiome interventions, which may have diverted attention from single-strain studies like those on LGG.

As understanding of the gut-lung axis deepens, the role and mechanisms of LGG in improving pediatric respiratory health are expected to become clearer. Future research directions for LGG are promising and multifaceted. First, precise studies integrating microbiome analysis, metabolomics, and immunology are needed to elucidate how LGG modulates respiratory health through gut microbial regulation. Second, novel formulations, such as engineered nasal sprays, inhalable probiotics (61), or advanced encapsulation technologies (62, 63), could further enhance LGG’s stability, targeted delivery, and maximal efficacy. These innovations would enable LGG to better adapt to diverse clinical application scenarios. Third, combining LGG with antiviral drugs, vaccines, or immunomodulators presents opportunities for synergistic therapeutic effects.

In conclusion, while LGG has demonstrated clear benefits, further research is essential to align its application with modern clinical and technological advancements. By leveraging cutting-edge technologies, addressing pediatric research challenges, and expanding knowledge of the gut-lung axis, LGG could continue to play a valuable role in improving respiratory health in children.
